# Supplementary material for: RNAhub—an automated pipeline to search and align RNA homologs with secondary structure assessment
Source: Nucleic Acids Res. 2025 Apr 29;53(W1):W496–502. doi: 10.1093/nar/gkaf342 (PMC12230734; doi:10.1093/nar/gkaf342)
Supplement: gkaf342_Supplemental_File [file gkaf342_supplemental_file.pdf]

# SUPPLEMENTAL FIGURES

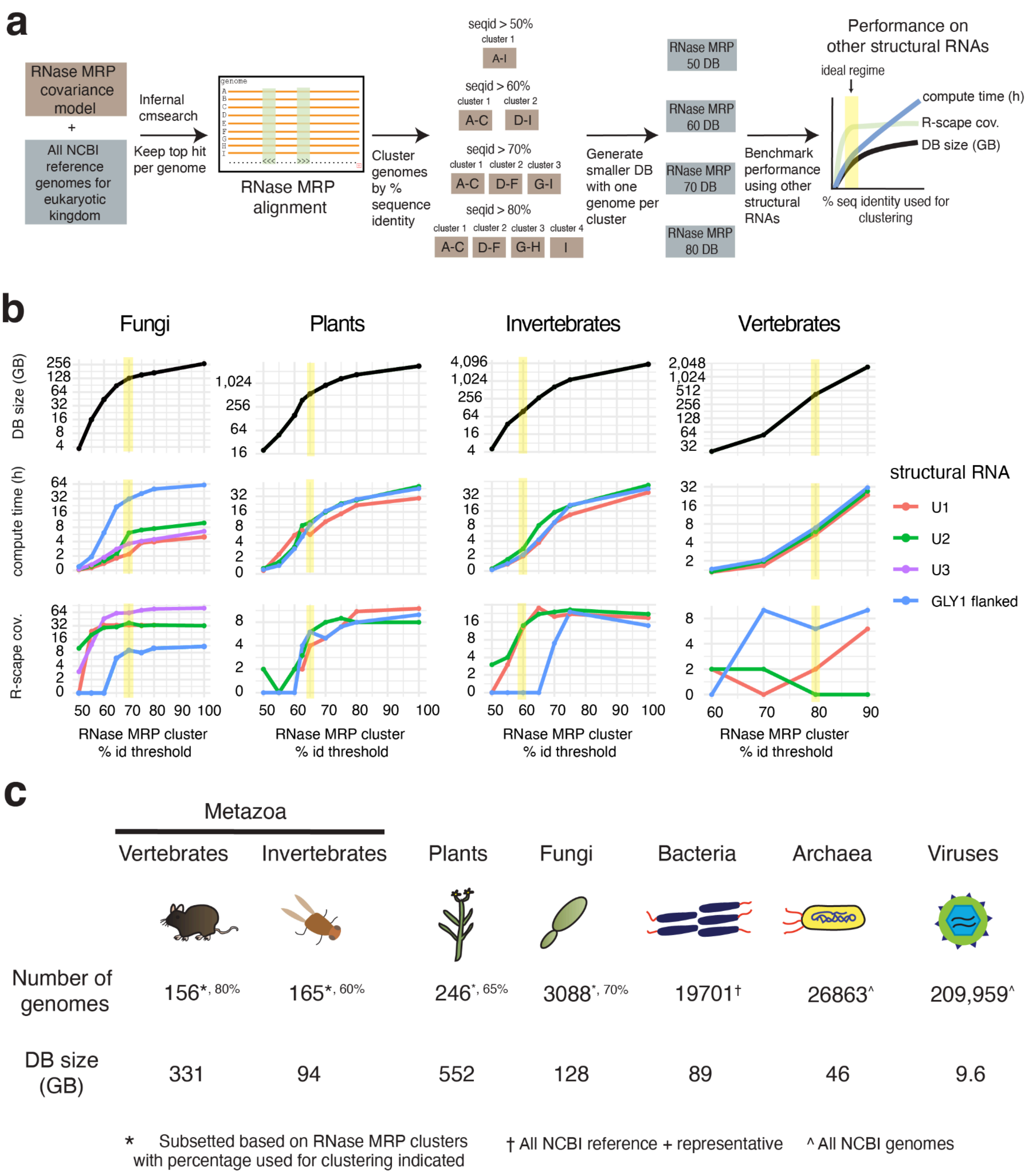

**Fig. S1**: Clustering of genomes **(a)** To reduce the size of eukaryotic databases, we clustered genomes based on their RNase MRP sequence (a highly conserved structural RNA), and kept one per cluster to construct smaller genome databases. We determined the "ideal regime" as the subclustered genome database that maximizes the tradeoff between the number of R-scape covariations vs. compute time and database (DB) size. **(b)** Database size, compute time, and R-scape covariation tradeoff (in log scale) for three non-RNase MRP structural RNAs using clustering thresholds ranging from 0.50 to 1.00, in increments of 0.05. The ideal regime that balances covariation against DB size and compute time is highlighted in yellow **(c)** Genome databases available in RNAhub, based on either the clustering at the ideal regime for eukaryotic genomes, or all reference NCBI for bacteria, and all NCBI archaea and viral genomes.
